# Oral Treatment with Plant-Derived Exosomes Restores Redox Balance in H_2_O_2_-Treated Mice

**DOI:** 10.3390/antiox12061169

**Published:** 2023-05-29

**Authors:** Rossella Di Raimo, Davide Mizzoni, Massimo Spada, Vincenza Dolo, Stefano Fais, Mariantonia Logozzi

**Affiliations:** 1 Department of Oncology and Molecular Medicine, Istituto Superiore di Sanità, 00161 Rome, Italy or rossella@exolabitalia.com (R.D.R.); davide.mizzoni@iss.itmassimo.spada@iss.it (M.S.); 2 ExoLab Italia, Tecnopolo d’Abruzzo, 67100 L’Aquila, Italy; 3 Department of Clinical Medicine, Public Health, Life and Environmental Sciences, University of L’Aquila, 67100 L’Aquila, Italy; vincenza.dolo@univaq.it

**Keywords:** plant-derived exosomes, antioxidants, anti-aging, anti-stress, natural bioactives, health, organic agriculture, plants

## Abstract

Plant-derived exosomes (PDEs) are receiving much attention as a natural source of antioxidants. Previous research has shown that PDEs contain a series of bioactives and that their content varies depending on the fruit or vegetable source. It has also been shown that fruits and vegetables derived from organic agriculture produce more exosomes, are safer, free of toxic substances, and contain more bioactives. The aim of this study was to investigate the ability of orally administered mixes of PDE (Exocomplex^®^) to restore the physiological conditions of mice treated for two weeks with hydrogen peroxide (H_2_O_2_), compared with mice left untreated after the period of H_2_O_2_ administration and mice that received only water during the experimental period. The results showed that Exocomplex^®^ had a high antioxidant capacity and contained a series of bioactives, including Catalase, Glutathione (GSH), Superoxide Dismutase (SOD), Ascorbic Acid, Melatonin, Phenolic compounds, and ATP. The oral administration of Exocomplex^®^ to the H_2_O_2_-treated mice re-established redox balance with reduced serum levels of both reactive oxygen species (ROS) and malondialdehyde (MDA), but also a general recovery of the homeostatic condition at the organ level, supporting the future use of PDE for health care.

## 1. Introduction

Reactive oxygen species (ROS) are mainly produced in mitochondria and are involved in various physiological activities, acting as both messengers of cell signaling and regulator of many cellular functions, including gene expression, proliferation, differentiation, and stress response [1]. The redox imbalance due to the absolute increase in ROS formation contributes to marked alteration of physiological functions [2], also leading to disease development [1,3,4]. These apparently contradictory effects are due to the imbalance between the oxidative and the reducing processes, a condition known as oxidative stress, inevitably leading to cell and tissue damage. In fact, oxidative stress is involved in the activation of a variety of transcription factors, in turn contributing to the activation of inflammatory pathways related to altered gene expression and often to the process of telomere shortening [5]. As a consequence, inflammation triggered by oxidative stress represents a major cause of many chronic pathologic conditions, including neurodegenerative diseases and cancers [1,6]. The role of oxidative stress in the aging process [7], induced by the progressive accumulation of ROS into the cells of the various organs and compartments of the body, is well-known, in turn leading to tissue damage and the loss of function associated with aging [6,8]. Currently, both prevention and cure of diseases for which a redox imbalance has a pathogenetic role are based on antioxidant strategies that aim to scavenge excessive ROS production and body accumulation [9,10]. In previous reports on C57BL/6J female mice, we have shown that daily oral intake of either alkaline water [11] or fermented papaya (FPP^®^) [12,13] both induced significantly anti-aging effects at the molecular level (e.g., telomere length) that was always associated with reduced ROS blood levels. The commercially available anti-oxidants are, to date, represented by a series of suppliers obtained from either chemical synthesis or alcoholic extractions, with a low level of bioavailability [14]. Currently, a critical discussion is focusing on the advantages and drawbacks of the extraction methods applied to foods and food derivates. In fact, biologically active compounds (polyphenols, tocopherols, organosulfur compounds, carotenoids, etc.) derived from various plant sources (fruits, vegetables, etc.) are mainly extracted through conventional solid–liquid extraction [14]. The conventional extraction approach markedly affects the quality of the obtained extracts for many reasons, including the thermal and the chemical treatments, which reduce both the yield and the quality of the biologically active compounds, with additional toxicity due to the chemical products used [15,16]. These problems also stand today because although attempts have been made to create innovative extraction methods that are more selective, faster, sustainable, and heat sensitive, they still do not allow obtaining amounts suitable for industrial purposes [17].

For the above reasons, we focused our research on natural sources of antioxidants obtained from fruits and vegetables without chemical extraction. To this purpose, we started our investigation on extracellular vesicles (EVs) from fruits and vegetables. 

While in the pioneer studies (between the end of the 1990s and the early 2000s), the interest was focused on the way EVs take part in some pathological conditions (e.g., cancer, inflammation, infectious diseases), currently, plant-derived EVs are considered crucial in interconnecting organs and compartments of our body, and interconnecting living beings between them (inter-kingdom connections). In this context, the presence of EVs in food is also being increasingly considered. Actually, the existence of EVs in plants is not controversial anymore, and the amount of scientific reports on the possible use of plant-derived exosomes (PDE) as a source of bioactives of crucial importance in health care is increasing day-by-day [18]. In the last decade, scientific reports have been supporting a general agreement on the evidence that PDEs contain a series of anti-oxidant agents (e.g., ascorbic acid, glutathione, superoxide dismutase, catalase) that are probably complexed within the PDE [18,19]. Moreover, the level of bioavailability and stability of the PDE content is increased by the lipid bilayer membrane of PDE that protect the bioactives from the external stimuli [18,19], including the oxidative stress [20,21]. The lipid bilayer membrane of PDEs protects their content by the quick oxidation and degradation that occurs to all foods after ingestion through both the gastric juice and the bile [18,19,22], thus further increasing the PDE bioavailability. Therefore, it appears mandatory that the PDE content, represented by proteins, lipids, antioxidants, mRNA, and microRNA, is naturally transported in a protected way in order to reach cellular targets and exert their functions [23]. These properties make PDE the ideal delivery system for communication between different species, such as the plant and the animal kingdoms, but also a natural approach to counteract diseases characterized by a redox imbalance [23,24,25,26,27,28,29,30,31,32,33,34,35,36,37,38,39,40,41,42,43,44]. Moreover, PDE may well be used in the daily integration of very precious bioactives in contributing to preventing human diseases, also with regenerative effects [20,21,34,35,45,46]. Recent reports suggest that the anti-oxidant apparatus contained in the PDE may represent a sort of immune system for the plants [47] but also that it may participate in maintaining the stability of the gut microbiota in humans [29,48,49]. Together with their use as suppliers, another important exploitation of PDE is for drug delivery, which may potentially cover all chemical and biological molecules without any evidence of intrinsic toxicity [18,19,22,45,50,51,52,53]. Lastly, PDEs are scalable for industrial use, their production being exclusively dependent on agricultural productivity. In this regard, we have previously shown that PDE derived from organic fruits had some key advantages compared to those obtained from conventional fruits, including a significantly higher amount of PDE and higher levels of antioxidants at an equal amount of juice [19]. However, we did not have in vivo data supporting a systemic anti-oxidant effect of PDE. Most of all, there are no studies that investigated both the anti-oxidant response and the general response of the body to the administration of PDEs. The aim of this study was to investigate the antioxidant effect of a mix of PDEs obtained from different plants, called Exocomplex^®^, in restoring an in vivo redox imbalance. To this purpose, we used a model of mice pretreated for 2 weeks with hydrogen peroxide (H_2_O_2_), resulting in a marked redox imbalance, and then treated orally by mixes of PDE. 

## 2. Materials and Methods

### 2.1. In Vivo Studies

All the studies were approved by the ethical committee of the Italian National Institute of Health (Rome, Italy) and were conducted in accordance with the current Italian Law (Law 26/2014), authorization No. 792/2017-PR (prot. D9997.49 27/06/2017), that regulates experiments in laboratory animals. In total, 30 C57BL/6J female mice between 16 and 20 g (4 weeks of age) were purchased from Charles River Laboratories Italia s.r.l. (Calco, Lecco, Italy) and housed in the animal facility of the Italian National Institute of Health. Mice had 10 and 14 h periods of light and darkness, respectively, and were housed in a different number of animal cages, depending on the experiment, with ad libitum mice chow (Mucedola, Settimo Milanese (MI), Italy) and water intake. A veterinarian responsible for animal welfare checked mice twice a week to monitor signs of sufferance, such as weight loss, decreased water and food consumption, poor hair coat, decreased activity levels, and tumor ulcerations, according to the guidelines for a correct laboratory practice and signs of poor quality of life.

Mice were divided into three groups, 10 mice/group: control group (H_2_O group); group treated with hydrogen peroxide (H_2_O_2_ group); and group pretreated with hydrogen peroxide and then treated with Exocomplex^®^ (Exocomplex^®^ group). The H_2_O group was not treated and drank only untreated water for the period of the experimentation (five weeks); the H_2_O_2_ group was treated with 1% hydrogen peroxide (H_2_O_2_) dissolved in water for two weeks, and then, the mice were watered with untreated water for three weeks. At the same time, the Exocomplex^®^ group was first treated with 1% hydrogen peroxide (H_2_O_2_) dissolved in water for two weeks, and then the mice were treated with Exocomplex^®^ for three weeks via gavage administration. We performed preliminary experiments in order to evaluate the potential toxicity of plant-derived exosomes (PDE) with different routes of administration (intraperitoneal, sublingual, and gavage), not showing toxicity with all the doses and with all the routes of administration used (not shown). Thus, we decided to use the maximal dose/mouse of Exocomplex^®^ (6 × 10^9^ PDE), corresponding to 2 × 10^8^ PDE/g mouse weight, dissolved in 200 μL of H_2_O, administered via gavage. Just before the mice sacrifice, blood was withdrawn from the mice’s eyes. Immediately after the sacrifice, bone marrow was isolated from both tibias and femurs of the mice’s hind legs; ovaries were retrieved from the reproductive system, while splenocytes were obtained from the spleen. Blood, bone marrow cells, ovarian germ cells, and splenocytes were used for subsequent experimental analyses of aging parameters.

### 2.2. Collection and Processing of Murine Plasma from Blood Samples

Blood sample collections from each group of mice were performed by retro-orbital bleeding (ROB) immediately before the sacrifice. This safe phlebotomy technique allowed to obtain the high-quality samples of adequate volume (500 μL/mouse) for analysis [54]. Blood samples were collected in eppendorf tubes and stored at room temperature for K3-EDTA-coated collection tubes. To obtain plasma samples, EDTA-treated whole blood from each mouse was centrifuged at 400× *g* for 20 min. Plasma samples (250 μL/mouse) were then collected and immediately analyzed or stored at −80 °C until the analysis.

### 2.3. Bone Marrow Cells Recovery from Mice

Immediately after the sacrifice of mice, bone marrow was obtained from both tibias and femurs of the hind legs [12]. Bone marrow was then placed in a physiological solution (sodium chloride, NaCl) and disrupted with the blunt end of a 5-mL syringe plunger. Bone marrow cells were isolated using a Falcon^®^ 100 μm cell strainer (Corning, New York, NY, USA), obtaining a uniform single-cell suspension from bone marrow. The single-cell suspensions were washed twice in PBS and immediately processed for the following analyses.

### 2.4. Ovarian Germ Cells Recovery from Mice

Immediately after the sacrifice of mice, ovaries were dissected [12], placed in physiological solution (NaCl) with 1% of trypsin and 0.1 μM of EDTA, separated from the remaining reproductive system with a cutter, and disrupted with the blunt end of a 5-mL syringe plunger. Ovarian germ cells were isolated using a Falcon^®^ 100 μm cell strainer (Corning, New York, NY, USA); connective tissue and debris were allowed to settle, obtaining a uniform single-cell suspension from ovarian tissue. The single-cell suspensions were washed twice in PBS and immediately processed for the following analyses.

### 2.5. Splenocytes Recovery from Mice

Immediately after the sacrifice of mice, splenocytes were obtained from the spleen. The spleen was then placed in physiological solution (NaCl) and disrupted with the blunt end of a 5-mL syringe plunger. Splenocytes were isolated and dissociated using a Falcon^®^ 100 μm cell strainer (Corning, New York, NY, USA), obtaining a uniform single-cell suspension from the spleen. The single-cell suspensions were washed twice in PBS and immediately processed for the following analyses. 

### 2.6. Exocomplex^®^

Exocomplex^®^ is a mix of plant-derived exosomes (PDE) that we purified and concentrated from fruits and vegetables from Italian organic agriculture. The result was a nanovesicle concentrate product containing 100% of the phytocomplex, active ingredients found in the plant of origin in a form immediately bioavailable to the body. The mixes were always obtained using the same proportions between different fruits and vegetables and using NTA as a quality control allowing an objective scalability of the product. Moreover, we performed all experimental controls by processing the mix under different conditions for numerous measurements, both intra-laboratory and between different operators, thus obtaining reproducibility and robustness of the measurements performed. The variability measured in the mean number of exosomes is 1.7%. Specifically, the fruits and vegetables from which the exosomes contained in the Exocomplex^®^ were asparagus (*Asparagus officinalis*), cherry (*Prunus avium* L.), grape (*Vitis vinifera* L.), kiwi (*Actinidia chinensis*), orange (*Citrus sinensis* (L.) Osbeck), blood orange (*Citrus sinensis* (L.) Osbeck “Blood Orange”), lemon (*Citrus limon* (L.) Osbeck), mango (*Mangifera indica* L.), papaya (*Carica papaya* L.), grapefruit (*Citrus paradisi Macfad*), bergamot (Citrus × bergamia Risso and Poit.), tomato (*Solanum lycopersicum* L.). All fruits and vegetables were purchased from several farms with organic farming certifications. The fruits and vegetables were subjected to different washing processes based on an initial washing with tap water, followed by washing with distilled water and sodium bicarbonate and rinsing with ultrafiltered water. Fruits (with peeled skin) and vegetables were extracted with a fruit juice extractor (Hurom Slow Juicer, HW Series, Hurom Co., Ltd., Gimhae-si, GYEONGSANGNAM-DO, Korea). Fruit and vegetable juices were stored at −80 °C in order to respect the seasonality of the organic agriculture products.

### 2.7. Exocomplex^®^ Isolation

Fruit juices were centrifuged at 500× *g* for 10 min at 10 °C; the supernatants were filtered with 100 µm filters and serially centrifugated at 2000× *g* for 20 min at 10 °C to eliminate cell debris and then at 15,000× *g* for 30 min at 10 °C to eliminate the fraction enriched in microvesicles. The supernatants were subsequently ultracentrifuged in a Sorvall WX Ultracentrifuge Series (Thermo Fisher Scientific, Waltham, MA, USA) at 110,000× *g* for 1 h 30 min at 10 °C to collect the Exocomplex^®^. The pellet was resuspended in ultra-filtered and sterilized water (or in PBS) for downstream analyses and preserved at +4 °C. 

### 2.8. Nanoparticle Tracking Analysis (NTA)

Nanoparticle Tracking Analysis (NTA) from Malvern (NanoSight NS300, Worcestershire, UK) was used for the measurement of size distribution and concentration of Exocomplex^®^ samples in the liquid suspension. Five videos of typical 60 s duration were taken. Data were analyzed using the NTA 3.0 software (Malvern Instruments), which was optimized to first identify and then track each particle on a frame-by-frame basis. The Brownian motion of each particle was tracked using the Stokes–Einstein equation: D° = kT/6π*η*r, where D° is the diffusion coefficient; kT/6π*η*r = f_0_ is the frictional coefficient of the particle, for the special case of a spherical particle of radius r moving at a uniform velocity in a continuous fluid of viscosity *η*; k is Boltzmann’s constant, and T is the absolute temperature [19,55,56,57].

### 2.9. Trypan Blue Cell Counting

After isolation, ovarian germ cells, bone marrow cells, and splenocytes of C57BL/6J female mice were counted by trypan blue exclusion under the optical microscope. Data were expressed as a number of cells.

### 2.10. Cell Proliferation Assay

Ovarian germ cells, bone marrow cells, and splenocyte proliferation was measured using Alkaline Phosphatase Yellow (pNPP) Liquid Substrate for ELISA (Sigma-Aldrich, St. Louis, MO, USA). The product is supplied as a ready-to-use buffered alkaline phosphatase substrate that contains p-nitrophenylphosphate (pNPP). According to the manufacturer’s protocols, the solution (100 µL/well) was added to cells seeded in a 96-well plate. Following the reaction with alkaline phosphatase, a yellow reaction product formed was read at 405 nm in a microplate reader (BioTek Epoch, Agilent, Santa Clara, CA, USA) [58]. Data are expressed as optical density × 1000 (arbitrary unit, a.u.). 

### 2.11. Transmission Electron Microscopy (TEM) of Exocomplex^®^

After isolation, Exocomplex^®^ was resuspended in PBS and properly diluted, and then incubated for 5 min onto carbon-coated copper grids, 200 mesh (Electron Microscopy Sciences, Hatfield, PA, USA) at room temperature. Once absorbed on the grids, Exocomplex^®^ was fixed with 2% glutaraldehyde in PBS (Electron Microscopy Sciences, Hatfield, PA, USA) for 10 min and then washed three times in Milli-Q water; negative staining was performed with 2% phosphotungstic acid; finally, the grids were air-dried and observed using a CM 100 Philips [56,59,60,61].

### 2.12. Total Antioxidant Power Assay (PAO Test Kit)

The detection and quantification of Total Antioxidant Capacity were performed in Exocomplex^®^ using a colorimetric assay, the PAO Test kit for Total Antioxidant Capacity (JaICA, Tokyo, Japan). The assay can detect not only hydrophilic antioxidants, such as Vitamin C and glutathione, but can also detect hydrophobic antioxidants, such as Vitamin E. The determination of the antioxidant power was carried out using a reduction in cupric ions (Cu^++^ to Cu^+^). Briefly, samples were incubated for 3 min at room temperature with a Cu^++^ solution, and the Cu^++^ was reduced by antioxidants to form Cu^+^ that reacts with a chromatic solution (bathocuproine) and can be detected by absorbance at a wavelength of 480 to 490 nm. Antioxidant capacity can be calculated from the Cu^+^ formed. Absorbance was recorded at 490 nm. Data were normalized per dose of Exocomplex^®^ administered to mice and expressed as mM.

### 2.13. Ascorbic Acid Assay

Detection and quantification of Ascorbic Acid in Exocomplex^®^ samples were performed using a fluorometric Ascorbic Acid Assay Kit (Sigma-Aldrich, St. Louis, MO, USA). Samples were diluted in an ascorbic acid buffer in a 96-well plate, and subsequently, a catalyst and then reaction mix was added to each well (the reaction mix is composed of an ascorbic acid buffer, ascorbic acid probe, and ascorbic acid enzyme mix). After 5 min of incubation, fluorescence was read in a microplate reader at Ex/Em = 535/587 nm. Data were normalized per dose of Exocomplex^®^ administered to mice and expressed as µg.

### 2.14. Catalase Activity Assay

For the Catalase Activity Assay (Abcam, Cambridge, UK), a fluorometric kit was used for the detection and quantification of the Catalase activity in Exocomplex^®^ samples. Briefly, samples resuspended in PBS were loaded in a 96-well plate; a stop solution was added in the control samples and incubated for 5 min at 25 °C to inhibit the Catalase activity. Catalase reaction mix (with H_2_O_2_) was added to both the control and high control samples for 30 min at 25 °C. The reaction in the high control samples and standard samples was stopped with the stop solution; then, the developer was added to all wells, and after 10 min, the fluorescence was read at Ex/Em = 535/587 nm on a micro-plate reader (Promega, Madison, WI, USA). Data were analyzed using the manufacturer’s instructions. One unit of Catalase corresponds to the amount of Catalase that will decompose 1 µmol of H_2_O_2_ per minute at pH 4.5 at 25 °C. Data were normalized per dose of Exocomplex^®^ administered to mice and expressed as mU/mL.

### 2.15. Reduced Glutathione (GSH) Detection and Quantification Assay

The Glutathione Colorimetric Detection Kit (Thermo Fisher Scientific, Waltham, MA, USA), a colorimetric assay, was used for the detection and quantification of reduced glutathione (GSH) levels in Exocomplex^®^ samples. Detection reagent and reaction mixture (NADPH and glutathione reductase) were added to samples, and after 20 min of incubation at room temperature, the optical densities were recorded at 405 nm. Data were normalized per dose of Exocomplex^®^ administered to mice and expressed as µM.

### 2.16. Superoxide Dismutase (SOD) Activity Assay

The Superoxide Dismutase Activity kit (Thermo Fisher Scientific, Waltham, MA, USA), a colorimetric assay, was used for the detection and quantification of the superoxide dismutase activity in Exocomplex^®^ samples. Samples were incubated for 20 min at room temperature after the addition of the sample and substrate, and chromogenic detection reagent. The optical densities were recorded at 450 nm. Data were normalized per dose of Exocomplex^®^ administered to mice and expressed as U/mL.

### 2.17. Total Reactive Oxygen Species (ROS) Assay 

Analysis of the total ROS levels was performed in preparations of the serum, bone marrow cells, and in splenocytes obtained from mice just before the sacrifice. For this purpose, a total reactive oxygen species (ROS) Assay Kit 520 nm (Thermo Fisher Scientific, Waltham, MA, USA) was used. An amount of 10 µL of plasma samples was added to 100 µL of 1× ROS Assay Stain. After 60 min of incubation at 37 °C and 5% CO_2_, signals were analyzed using a fluorescent microplate reader off the 488 nm (blue laser) in the FITC channel. Data are expressed as Median Intensity of Fluorescence (M.F.I.), arbitrary unit (a.u.).

### 2.18. Detection of Telomeres by PNA Kit/FITC for Flow Cytometry 

Detection of telomeres was performed in ovarian germ cells of mice obtained immediately after the sacrifice. To this purpose, a Telomere PNA Kit/FITC for Flow Cytometry (Dako—Agilent, Santa Clara, CA, USA) was used. The kit allows the detection of telomeres in nucleated hematopoietic cells using a fluorescence in situ hybridization and a fluorescein-conjugated peptide nucleic acid (PNA) probe. Results are evaluated by flow cytometry using a light source with excitation at 488 nm. Data are expressed as M.F.I. (a.u).

### 2.19. Lipid Peroxidation (MDA) Assay Kit

Lipid peroxidation was analyzed in murine serum as a marker of oxidative stress to evaluate the degradation of lipids (above all polyunsaturated lipids) as a result of oxidative damage, resulting in the production of malondialdehyde (MDA). Lipid peroxidation was measured by using the Lipid peroxidation (MDA) Assay Kit (Sigma-Aldrich, MO, USA) by the reaction of MDA with thiobarbituric acid (TBA) to form a fluorometric (Ex = 532/Em = 553 nm) product, proportional to the MDA present. The amount of MDA present in the samples was determined from the standard curve. Data are expressed as M.F.I. (a.u).

### 2.20. Freeze-Drying of Exocomplex^®^ Samples

Exocomplex^®^ samples were lyophilized by using the freeze-drying technique with the freeze-dryer (Hyper-COOL system, Sorisole (BG), Italy). Lyophilization is a dehydration technique using the sublimation process, the shift from the solid state (completely frozen) directly into the gas without passing through the liquid phase. Additionally, applying a vacuum enables lowering the pressure below the triple point, which avoids the liquid phase. After the purification process, 50 mM of final trehalose concentration was added to the plant-derived nanovesicles in liquid form. The samples were frozen for 24 h to −80 °C and lyophilized for 72 h. The lyophilized samples were resuspended in H_2_O for downstream analyses.

### 2.21. DNA Damage Assay Kit

DNA damage was detected and quantified in murine siero by using DNA Damage Competitive ELISA Kit (Invitrogen, Thermo Fisher Scientific, Waltham, MA, USA), a solid-phase competitive Enzyme-Linked Immunoassorbent Assay (ELISA) confirmed that all three oxidized guanine species, 8-hydroxy-2′-deoxyguanosine (8-OHdG) from DNA, 8-hydroxyguanosine from RNA, and 8-hydroxyguanine from digested DNA, were from DNA or RNA. Among numerous types of oxidative DNA damage, the formation of 8-hydroxy-2′-deoxyguanosine (8-OHdG) is a ubiquitous marker of oxidative stress. Standards or diluted samples were added into a clear microtiter plate coated with an antibody to capture rabbit antibodies. An 8-hydroxyguanosine conjugate was added to the standards and samples in the wells. The binding reaction was initiated by the addition of a peroxidase-labeled mouse monoclonal antibody to 8-hydroxy-2′-deoxyguanosine in each well. After a 1-h incubation, the plate was washed and substrate added. The substrate reacts with the peroxidase-labeled antibody that has reacted with the bound conjugate. After a short incubation, the reaction was stopped, and the absorbance was read at 450 nm in a microtiter plate. Data are expressed as ng/mL of 8-OHdG.

### 2.22. Mitochondrial Membrane Potential Measurement

Mitochondrial membrane potential was measured in bone marrow cells and splenocytes of mice (isolated immediately after the sacrifice) using MitoTracker^®^ Mitochondrion-Selective Probes (Molecular Probes, Invitrogen, Thermo Fisher Scientific, Waltham, MA, USA), a green-fluorescent mitochondrial stain, which appears to localize to mitochondria regardless of mitochondrial membrane potential. To label mitochondria, live cells were incubated with 100 nM of MitoTracker^®^ probes, which passively diffused across the plasma membrane and accumulated in active mitochondria. The reduced probes do not fluoresce until they enter live cells, where they are oxidized to the corresponding fluorescent mitochondrion-selective probe and then sequestered in the mitochondria. Suspension cells were centrifuged to obtain a cell pellet that was resuspended in a prewarmed (37 °C) MitoTracker^®^ probe staining solution. After 30 min of incubation at 37 °C and 5% CO_2_, the cells were re-pelleted by centrifugation and resuspended in a fresh prewarmed medium or buffer. Green fluorescence was read at Ex/Em = 490/516 nm in a microplate reader. Data are expressed as M.F.I. (a.u.).

### 2.23. Mitochondrial Superoxide Detection

Mitochondrial superoxide was measured in bone marrow cells and splenocytes of mice (isolated immediately after the sacrifice) using Mitochondrial Superoxide Detection Kit (Abcam, Cambridge, UK), a sensitive fluorometric one-step assay based on MitoROS 580 dye to detect intracellular superoxide radical in live cells. The dye is cell-permeable and selectively reacts with mitochondrial superoxide present in live cells to generate a red fluorescence signal that was read at Ex/Em = 540/590 nm in a microplate reader after incubation at 37 °C for 60 min. The results were calculated as fluorescence intensity difference between control and treated cells. Data are expressed as M.F.I. (a.u.).

### 2.24. ATP Assay Kit

Total ATP was measured in Exocomplex^®^ samples with the ATP Assay Kit (Colorimetric) (Abcam, Cambridge, UK). The assay was based on the phosphorylation of glycerol in order to generate a product that was quantified colorimetrically (OD 570 nm). After plating standard wells, sample wells, and sample background control wells at the optimal dilution, the reaction mix was added to each standard and sample well, and the background reaction mix was added to the background control sample wells. The samples were incubated at room temperature for 30 min, protected from light, and the absorbance was measured on a microplate reader at OD 570 nm. Finally, the standard curve was constructed, and the ATP concentration (nmol/µL or µmol/mL or mM) was calculated with the following formula:

ATP concentration = (B/V*D)*DDF, where B = amount of ATP in the sample well calculated from the standard curve (nmol or mM); V = sample volume added in the sample wells (µL); D = sample dilution factor if the sample is diluted to fit within the standard curve range (prior to the reaction well setup; DDF = deproteinization dilution factor. Data were normalized per dose of Exocomplex^®^ administered to mice and expressed as µM of ATP.

### 2.25. Melatonin Determination in Serum Samples

The melatonin concentration was measured in serum samples using the colorimetric Melatonin ELISA Kit (Novus Biologicals, Centennial, CO, USA). The optical density was read at 450 nm in a microplate reader. The standard curve was constructed, and the melatonin concentration (ng/mL) was calculated. 

### 2.26. Serotonin Determination in Serum and Urine Samples

The serotonin concentration was measured both in serum and urine samples using the colorimetric Serotonin ELISA Kit (Novus Biologicals, Centennial, CO, USA). The optical density was read at 405 nm in a microplate reader. The standard curve was constructed, and the serotonin concentration (ng/mL) was calculated. The intensity of the yellow coloration is inversely proportional to the amount of serotonin captured in the plate.

### 2.27. Total Ig Detection and Quantification in Serum Samples

Protein levels of total Ig were detected and quantified in serum samples using IgG (Total) Mouse Uncoated ELISA Kit with Plates and IgA Mouse Uncoated ELISA Kit with Plates (Invitrogen, Thermo Fisher Scientific, Waltham, MA, USA). The optical density values were read at 450 nm in a microplate reader; the standard curve was constructed, and the concentration of total Ig (µg/mL) was calculated.

### 2.28. Statistical Analysis

Results are reported as means ± standard error (SE), calculated using the GraphPad Prism software (San Diego, CA, USA). Statistical significance was set at *p* < 0.05. The statistical analysis was conducted with one-way ANOVA Bonferroni.

## 3. Results

### 3.1. Quantitative and Qualitative Analysis of the Exocomplex^®^

#### 3.1.1. The Bioactives’ Content

We used a patented technology platform based on mixing exosomes from different fruits and vegetables aimed at creating the products with a specific indication as a supplier (Exocomplex^®^). (Italian Patent No. 102021000001343, Granted PCT/IB2022/050587) [62] Therefore, this first set of experiments was aimed at preparing a mix of exosomes deriving from different fruits and vegetables in order to obtain a pull of natural antioxidants with multiple targets. To this purpose, we purified and concentrated plant-derived exosomes from different fruits and vegetables derived from Italian organic farming. 

The fruits and vegetables were subjected to different washing procedures as described in M and M, and the extracts and juices were stored at −80 °C.

We, thus, characterized and quantified the bioactive molecules and the antioxidants contained in the whole mix. We found high levels of SOD-1, GSH, catalase, ascorbic acid, melatonin, phenolic compound, and ATP, together with a very high total antioxidant capacity (Table 1).

#### 3.1.2. Concentration and Size-Distribution

**Figure 1:**
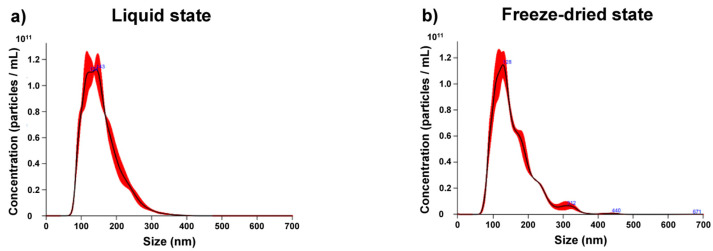
Analysis of concentration and size distribution of Exocomplex^®^ by Nanoparticle tracking analysis (NTA). (**a**) NTA profile of Exocomplex^®^ in the liquid state. (**b**) NTA profile of Exocomplex^®^ in the freeze-dried state.

Then, we evaluated the quality of the Exocomplex^®^ preparation by Nanoparticle Tracking Analysis (NTA) that shows both the amount and the size distribution of the vesicles contained in the sample. The analysis was performed both when the sample was in a liquid phase and after the freeze-drying of the same sample. Briefly, the sample was frozen with a cryogenic protector (trehalose) at the final concentration of 50 mM at −80 °C and subjected to the freeze-drying process; the lyophilized was then manually pulverized with a mortar, resuspended in water, and analyzed by NanoSight for concentration and size distribution (Figure 1). The results showed that the freeze-drying process did not interfere with either the number or the size of the exosomes contained in the sample (Figure 1).

#### 3.1.3. Morphological Characterization

**Figure 2:**
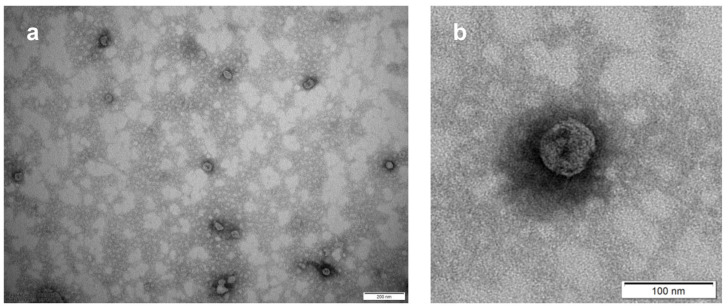
Transmission electron microscopy (TEM) of Exocomplex^®^. (**a**) Typical rounded structure and nanometer size of Exocomplex^®^. (**b**) Structural integrity of Exocomplex^®^.

To further verify the fitness level of our Exocomplex^®^ samples, we used transmission electron microscopy (TEM). TEM analysis clearly showed the presence of numerous typically rounded, whole, and undamaged small vesicles with a size largely under 200 nm, suggesting their exosome-like nature (Figure 2a). A higher magnification confirmed the integrity of the exosome-like nanovesicles showing the presence of an unbroken bilayer membrane, visible as a thin white filament surrounding the electron-dense exosome content (Figure 2b). Thus, our Exocomplex^®^ contained nanosized vesicles, showing the typical shape of the exosomes and with an intact external membrane.

### 3.2. In Vivo Evaluation of the Exocomplex^®^ in Mice Treated with H_2_O_2_

This set of experiments was aimed at verifying the ability of the Exocomplex^®^ to restore a redox balance in mice treated with H_2_O_2_. To this purpose, mice were treated with H_2_O_2_ for two weeks and then either treated with water only (H_2_O_2_ group) or treated orally by Exocomplex^®^ (Exocomplex^®^ group). Both groups were compared to mice that received water only (H_2_O group). At the sacrifice, blood samples and the organs were obtained from each mouse and analyzed for the redox balance and various cellular functions.

#### 3.2.1. Antioxidant Effect of Exocomplex^®^ on Serum ROS Levels In Vivo Treatment

**Figure 3:**
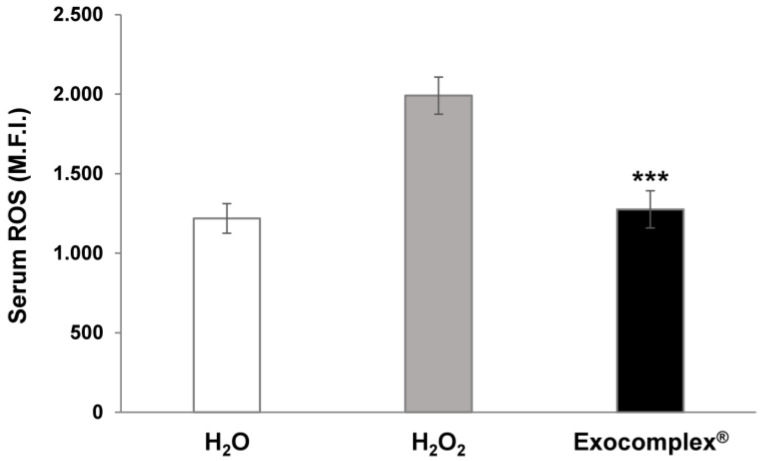
Serum ROS levels in C57BL mice after treatment with Exocomplex^®^. Analysis of the total ROS levels (Mean Fluorescent Intensity, M.F.I.) was performed on the serum of blood samples collected just before the sacrifice of the mice. Analysis was performed with a fluorimetric assay, and the signals emitted were measured on a microplate reader at 488 nm (blue laser) in the FITC channel. Data are expressed as means ± SE. *** *p* < 0.001.

The first analysis was to measure the ROS plasmatic levels by comparing mice treated with H_2_O_2_ alone and those receiving Exocomplex^®^ after the H_2_O_2_ treatment. The results have shown that the ROS plasmatic levels were significantly increased in the H_2_O_2_ group (1990 ± 117 arbitrary unit, a.u., *p* < 0.0001, Standard deviation (SD) = ±371) compared to the mice receiving only water (1218 ± 94 a.u., SD = ±296) while the Exocomplex^®^ treatment dramatically reduced the ROS plasmatic levels in the H_2_O_2_ (1276 ± 117 a.u., *p* < 0.001, SD = ±370), restoring the condition of the mice receiving water only from the beginning of the experiments (Figure 3). This result strongly supported a systemic anti-oxidative effect of the Exocomplex^®^ in curing a relevant redox imbalance induced by H_2_O_2_. 

#### 3.2.2. Antioxidant Effect of Exocomplex^®^ on Serum Lipid Peroxidation In Vivo Treatment

**Figure 4:**
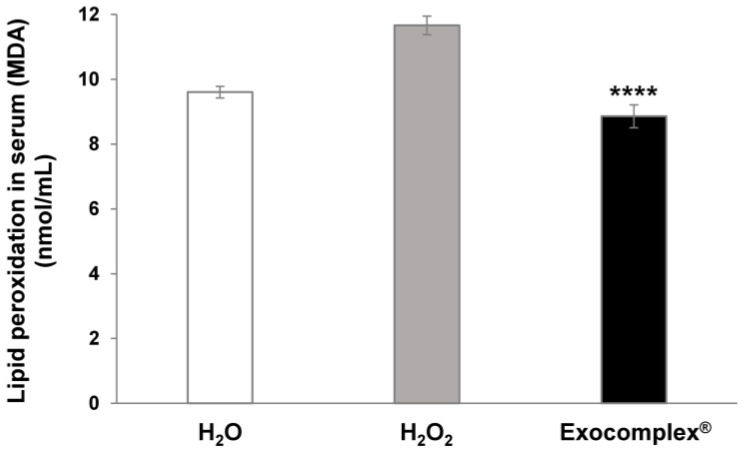
Serum lipid peroxidation measurement in C57BL mice after treatment with Exocomplex^®^. Quantification of the lipid peroxidation was performed on the serum of blood samples collected just before the sacrifice of the mice. Lipid peroxidation was evaluated through the concentration of malondialdehyde (MDA) (nmol/mL), resulting from oxidative damage. Analysis was performed with a fluorimetric assay, and the relative fluorescence units (RFU) were measured at Ex = 532 nm/Em = 553 nm on a microplate reader (green laser). Data are expressed as means ± SE. **** *p* < 0.0001.

We knew that ROS interacted with lipids and oxidized unsaturated lipid chains, leading to the formation of a hydroperoxidized lipid and an alkyl radical. Moreover, lipid peroxides are also able to propagate further generation of ROS or degrade into reactive compounds capable of crosslinking DNA and proteins. Malondialdehyde (MDA) is one of the final products of polyunsaturated fatty acids peroxidation, and higher production of MDA is correlated to increased ROS concentration [63]. For the above reason, in the same blood samples, we measured the MDA levels. The results showed that the serum concentration of MDA in mice subjected to oxidative stress (H_2_O_2_ group) was significantly higher (11.7 ± 0.3 nmol/mL, *p* < 0.0001, SD = ±0.9 nmol/mL) than in untreated mice (9.6 ± 0.2 nmol/mL, SD = ±0.6 nmol/mL) while mice receiving the Exocomplex^®^ after the H_2_O_2_ treatment showed significantly lower MDA levels (8.9 ± 0.4 nmol/mL, *p* < 0.0001, SD = ±1.1 nmol/mL) and comparable to the untreated mice (Figure 4). 

This set of results showed that consistent with the ROS serum levels, Exocomplex^®^ treatment significantly reduced the toxic effects of oxidative stress, leading to a marker of lipid peroxidation, such as MDA, to the physiological values shown in the untreated and unstressed samples.

#### 3.2.3. Effect of Exocomplex^®^ Treatment on Cell Number and Cell Proliferation

**Figure 5:**
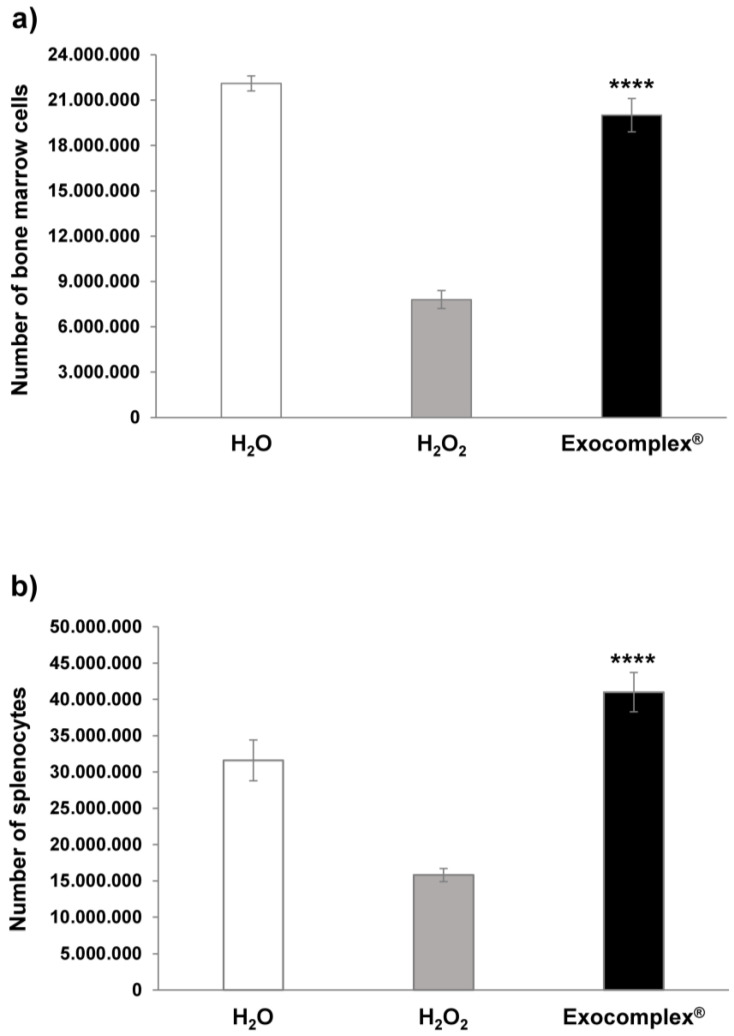
Number of bone marrow cells and splenocytes after treatment with Exocomplex^®^. Effect of Exocomplex^®^ treatment on the number of bone marrow cells and splenocytes obtained just after the sacrifice of the mice. The cells were counted by trypan blue exclusion under an optical microscope. (**a**) Number of bone marrow cells. (**b**) Number of Splenocytes. Data are expressed as means ± SE. **** *p* < 0.0001.

ROS, together with being the markers and the main agents of oxidative stress, are also signaling molecules involved in the regulation of many biological and physiological processes [64]. In fact, ROS are implicated in modulating cell proliferation, in the induction of apoptosis, contributing to cell senescence, and in either favoring or inhibiting immune response [65,66]. With this background, we performed a series of analyses on cells isolated from the femoral bone marrow and spleen of mice. We first analyzed simply the number of cells obtained from the various organs in the different conditions. The results showed that the H_2_O_2_ treatment induced a marked reduction in the cells isolated from both the bone marrow ((7.8 ± 0.6) × 10^6^ cells, *p* < 0.0001, SD = ±1.9 × 10^6^ cells, Figure 5a) and the spleen ((15.8 ± 0.9) × 10^6^ cells, *p* < 0.0002, SD = ±2.8 × 10^6^ cells, Figure 5b), compared to untreated mice receiving H_2_O only (No. bone marrow cells: (22.1 ± 0.5) × 10^6^ cells, SD = ±1.6 × 10^6^ cells; No. splenocytes: (31.6 ± 2.8) × 10^6^ cells, SD = ±8.8 × 10^6^ cells). The Exocomplex^®^ oral treatment restored the number of cells obtained from the bone marrow ((20 ± 1.1) × 10^6^ cells; *p* < 0.0001, SD = ±3.5 × 10^6^ cells, Figure 5a) while increasing the number of splenocytes ((41 ± 2.7) × 10^6^ cells; *p* < 0.0001, SD = ±8.5 × 10^6^ cells, Figure 5b) compared to the group receiving H_2_O_2_ only, and the mice that received water only. Thus, Exocomplex^®^ treatment, together with restoring redox balance, contributed to renewing the cellularity in immune organs, such as the bone marrow and the spleen. 

**Figure 6:**
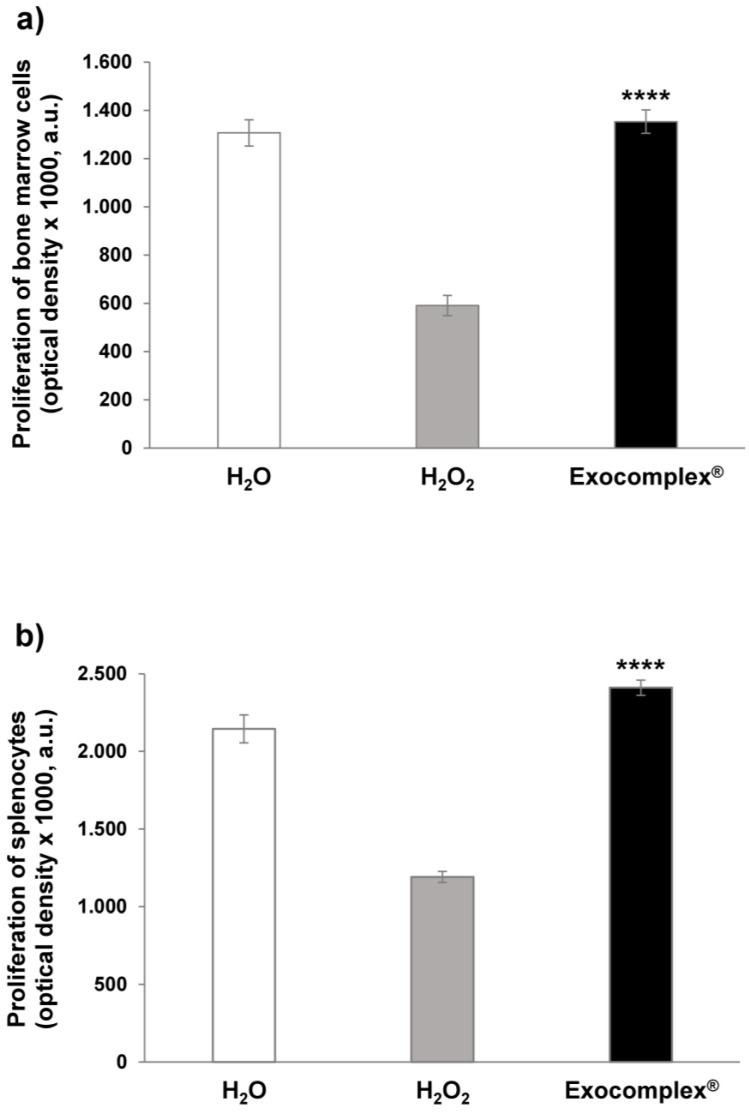
Proliferation of bone marrow cells and splenocytes after treatment with Exocomplex^®^. Effect of Exocomplex^®^ treatment on the proliferation of bone marrow cells and splenocytes obtained just after the sacrifice of the mice. The optical density values (a.u.) were read at 405 nm in a microplate reader after the reaction with alkaline phosphatase. (**a**) Proliferation of bone marrow cells. (**b**) Proliferation of Splenocytes. Data are expressed as means ± SE. **** *p* < 0.0001.

The following set of experiments was performed with the aim of verifying whether the increase in the cellularity after the Exocomplex^®^ treatment was due to a real increase in the proliferative capacity of the splenocytes and the bone marrow cells. To this purpose, we analyzed the spontaneous proliferation in cells isolated from bone marrow and spleen in mice treated with H_2_O_2_ with or without Exocomplex^®^ treatment and in mice that received water only from the beginning of the experiment. Consistently with the cell count, the H_2_O_2_ oxidative stress induced a marked decrease in proliferation in both bone marrow (591.3 ± 42.0 a.u.; *p* < 0.0001, SD = ±132.8 a.u., Figure 6a) and splenocytes (1191.0 ± 35.4 a.u.; *p* < 0.002, SD = ±111.8 a.u., Figure 6b) compared to the group that exclusively received H_2_O (bone marrow: 1306.5 ± 54.7 a.u., SD = ±172.8 a.u.; splenocytes: 2145.0 ± 90.1 a.u., SD = ±285.0 a.u.). The group of mice receiving Exocomplex^®^ after the H_2_O_2_ treatment showed significantly higher optical densities (bone marrow: 1353.3 ± 48.6 a.u.; *p* < 0.0001, SD = ±153.7 a.u., Figure 6a; splenocytes: 2410.5 ± 48.6 a.u., *p* < 0.0001, SD = ±153.7 a.u., Figure 6b) compared to H_2_O_2_ group; again, the splenocytes showed increased proliferation when compared to the control group. These results, on the one hand, showed that the increased cellularity was due to an increased proliferation capacity; on the other hand, they suggested that the Exocomplex^®^ treatment could induce an immune stimulation, in turn suggesting the use of plant-derived exosomes as an adjuvant treatment in immunostimulation protocols [67,68].

#### 3.2.4. Exocomplex^®^ Treatment Reduces Oxidative Stress in Mitochondria of Bone Marrow Cells and Splenocytes

**Figure 7:**
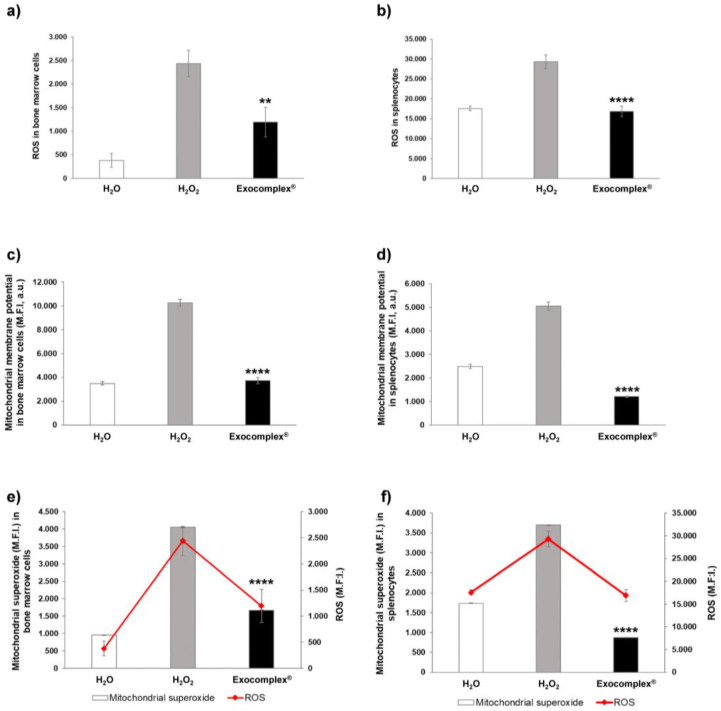
Effect of Exocomplex^®^ treatment on oxidative stress in bone marrow cells and splenocytes. (**a**) ROS levels (M.F.I., a.u.) in bone marrow cells measured in a fluorescence microplate reader at 488 nm (blue laser). (**b**) ROS levels (M.F.I., a.u.) in splenocytes measured in a fluorescence microplate reader at 488 nm (blue laser). (**c**) Mitochondrial membrane potential (M.F.I., a.u.) measurement in bone marrow cells. Green fluorescence values were read at Ex/Em = 490/516 nm in a fluorescence microplate reader. (**d**) Mitochondrial membrane potential (M.F.I., a.u.) measurement in splenocytes. Green fluorescence values were read at Ex/Em = 490/516 nm in a fluorescence microplate reader. (**e**) Comparison between the mitochondrial superoxide (M.F.I., a.u.) levels and ROS levels (M.F.I., a.u.) measured in bone marrow cells. Red fluorescence values related to mitochondrial superoxide were read at Ex/Em = 540/590 nm in a fluorescence microplate reader. Green fluorescence values related to ROS were read at 488 nm in a fluorescence microplate reader. (**f**) Comparison between the mitochondrial superoxide (M.F.I., a.u.) levels and ROS levels (M.F.I., a.u.) measured in splenocytes. Red fluorescence values related to mitochondrial superoxide were read at Ex/Em = 540/590 nm in a fluorescence microplate reader. Green fluorescence values related to ROS were read at 488 nm in a fluorescence microplate reader. Data are expressed as means ± SE. ** *p* < 0.01, **** *p* < 0.0001.

We, thus, wanted to investigate the effect of Exocomplex^®^ against oxidative stress at the cellular level. To this purpose, we evaluated ROS levels and mitochondrial damage in bone marrow cells and splenocytes obtained from the mice treated in the above-detailed way (Figure 7). First, as expected, the H_2_O_2_ treatment induced a strong increase in ROS accumulation in both bone marrow cells (2438.4 ± 278.2 a.u., *p* < 0.0001, SD = ±879.6 a.u., Figure 7a) and the splenocytes (29,290.6 ± 1375.4 a.u.; *p* < 0.0001, SD = ±5487.7 a.u., Figure 7b) compared to the mice that received H_2_O only (bone marrow: 378.9 ± 143.1 a.u., SD = ±452.7 a.u.; splenocytes: 17,576.9 ± 539.8 a.u., SD = ±1706.9 a.u.). Exocomplex^®^ treatment dramatically reduced the redox imbalance in mice that underwent H_2_O_2_-induced stress; this passed through a significant decrease in ROS levels in both bone marrow cells (1193.2 ± 315.4 a.u.; *p* < 0.01, SD = ±997.3 a.u., Figure 7a) and the splenocytes (16,874.0 ± 1286.9 a.u.; *p* < 0.0001, SD = ±4069.4 a.u., Figure 7b). To further support the above data, we evaluated the mitochondrial stress through the measurement of mitochondrial membrane potential and mitochondrial superoxide analyses. Monitoring of mitochondrial membrane potential is commonly used as an indicator of either cell health or cell injury, being related to the cells’ capacity to generate ATP by oxidative phosphorylation [69,70,71]. Mitochondrial membrane potential (ΔΨm) is not a stable value, being a dynamic parameter depending on the state of activation/differentiation of the cell; thus, if it undergoes a prolonged perturbation, it may compromise the viability of the cells, leading to cell death [70,71,72]. However, there is much evidence showing that at high ΔΨm, the mitochondrial respiratory chain becomes a potent producer of ROS; therefore, the generation of ROS is exponentially related to ΔΨm [71,73,74,75]. In our experiments, H_2_O_2_ induced an increase in mitochondrial membrane potential (bone marrow cells: 10,242.9 ± 294.4 a.u., *p* < 0.0001, SD = ±931.0 a.u., Figure 7c; splenocytes: 5052.9 ± 173.2 a.u., *p* < 0.0001, SD = ±547.6 a.u., Figure 7d) compared to mice that received H_2_O only. Splenocytes and bone marrow cells from mice that received Exocomplex^®^ after H_2_O_2_ treatment showed significantly lower ΔΨm (bone marrow: 3734.7 ± 260.3 a.u., *p* < 0.0001, SD = ±823.2 a.u., Figure 7c; splenocytes: 1206.3 ± 24.2 a.u., *p* < 0.0001, SD = ±76.6 a.u., Figure 7d) in comparison to the H_2_O_2_ group, thus restoring the physiological condition (bone marrow: 3486.6 ± 150.6 a.u., SD = ±476.2 a.u.; splenocytes: 2489.1 ± 88.7 a.u., SD = ±280.6 a.u.). Mitochondrial superoxide is a highly reactive molecule, usually converted in water and oxygen by the cellular antioxidant system, often overcoming the cellular antioxidant pathways and leading to proteins, lipids, and DNA damage [76]. For the above reasons, it is considered a major cause of cellular damage induced by oxidative stress. We measured the mitochondrial superoxide in the same cells by comparing the various mice groups. The results have shown that the mitochondrial superoxide levels were significantly higher in mice treated with H_2_O_2_ compared to mice receiving H_2_O only (bone marrow cells: 4050 ± 11 M.F.I., a.u., *p* < 0.0001, SD = ±34.5 a.u., Figure 7e; splenocytes: 3697.1 ± 1.9 a.u., *p* < 0.0001, SD = ±6.0 a.u., Figure 7f). Again, Exocomplex^®^ treatment induced a significant reduction in mitochondrial superoxide (bone marrow cells: 1668.4 ± 4.3 a.u., *p* < 0.0001, SD = ±13.5 a.u., Figure 7e; splenocytes: 870.8 ± 0.6 a.u., *p* < 0.0001, SD = ±2.0 a.u., Figure 7f), reaching values comparable to those measured in control H_2_O group (bone marrow cells: 958.9 ± 6.0 a.u., SD = ±19.1 a.u.; splenocytes: 1734.9 ± 3.0 a.u., SD = ±9.4 a.u.). 

All in all, this set of experiments showed that the Exocomplex^®^ treatment efficiently restored ROS levels, mitochondrial membrane potential, and mitochondrial superoxide induced by H_2_O_2_ treatment, reaching levels comparable to the control mice. We know that mitochondrial ROS promote cellular senescence and contribute to aging, by promoting mitochondrial oxidative stress [7,77], in turn suggesting that treatment with Exocomplex^®^ could be used in counteracting aging.

#### 3.2.5. Exocomplex^®^ Restores Physiological Concentration of Serum Immunoglobulins

**Figure 8:**
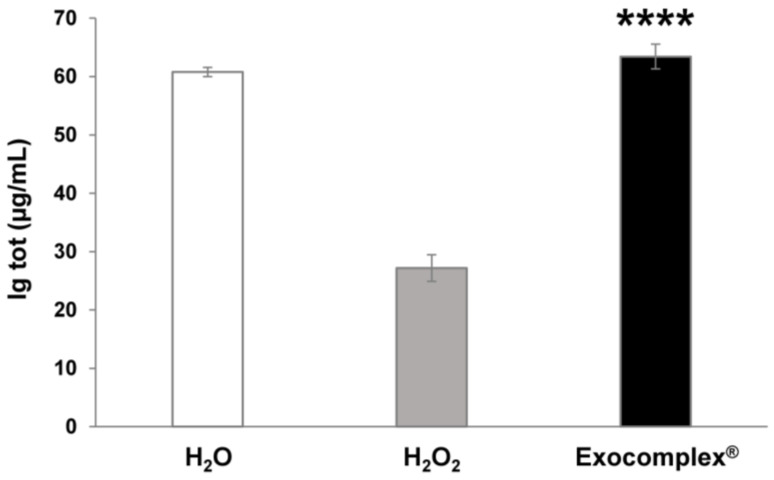
Effect of Exocomplex^®^ treatment on Ig tot expression. Protein levels of total Ig were detected and quantified on the serum of blood samples collected just before the sacrifice of the mice. The optical density values were read at 450 nm in a microplate reader, and the concentration of total Ig (µg/mL) was calculated from the standard curve. Data are expressed as means ± SE. **** *p* < 0.0001.

Recent reports suggest that plant-derived exosomes isolated from edible plants modulate inflammatory and immune responses [20,45,78,79]. On the other hand, exposure to oxidative stress significantly affected serum immunoglobulin levels [80,81]. Thus, we wanted to evaluate the impact of oxidative stress and Exocomplex^®^ treatment on immunoglobulin serum levels. To this purpose, we measured the Ig serum levels in the various mice groups by ELISA. Immunoglobulins serum levels were significantly reduced in H_2_O_2_-treated mice (27.2 ± 2.3 ng/mL, *p* < 0.0001, SD = ±11.4 ng/mL) compared to mice that received H_2_O only (60.8 ± 0.8 ng/mL, SD = ±3.9 ng/mL) (Figure 8); while mice treated with Exocomplex^®^ following H_2_O_2_ administration showed significantly higher immunoglobulins concentration (63.4 ± 2.1 ng/mL, *p* < 0.0001, SD = ±10.6 ng/mL) compared to the H_2_O_2_ group, leading to serum immunoglobulin levels comparable to the control group (H_2_O group) (Figure 8). 

This set of results suggests that Exocomplex^®^ treatment can efficiently contrast and balance the toxic effect of H_2_O_2_ treatment, directly influencing immunoglobulins serum concentration.

#### 3.2.6. Exocomplex^®^ Administration Balances Cytotoxic Effect Induced by Oxidative Stress

**Figure 9:**
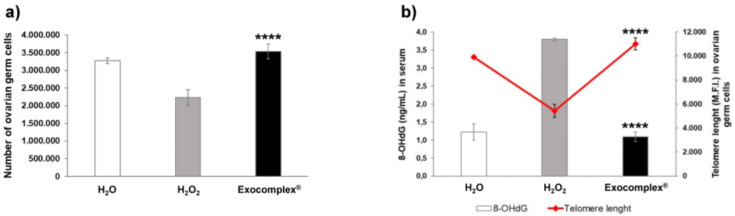
Effect of Exocomplex^®^ treatment on the oxidative stress in ovarian germ cells and in blood. Ovarian germ cells and serum of blood samples were obtained right after and before the sacrifice of the mice, respectively. (**a**) Number of ovarian germ cells. The cells were counted by trypan blue exclusion under an optical microscope. (**b**) Comparison between the DNA damage in serum and telomere length in ovarian germ cells. DNA damage was measured by the formation of 8-hydroxy-2′-deoxyguanosine (8-OHdG), a ubiquitous marker of oxidative stress. The absorbance was read at 450 nm in a microplate reader, and the concentration of 8-OHdG (ng/mL) was calculated from the standard curve. Telomere length was measured as fluorescence emission by flow cytometry after excitation at 488 nm. Data are expressed as means ± SE. **** *p* < 0.0001.

This set of experiments was aimed at investigating the effects of Exocomplex^®^ on other body functions that might suffer heavy redox imbalance, such as the ovarian germ line. We, thus, compared the cellularity and the telomere length in the different mice groups. As reported in Figure 9a, H_2_O_2_ induced a marked reduction in ovarian germ cells isolated from murine ovaries ((2.23 ± 0.22) × 10^6^ cells, *p* < 0.002, SD = ±0.71 × 10^6^ cells) compared to untreated H_2_O group ((3.27 ± 0.08) × 10^6^ cells, SD = ±0.24 × 10^6^ cells), while treatment with the Exocomplex^®^ treated mice showed a cell count comparable to H_2_O group ((3.53 ± 0.21) × 10^6^ cells, SD = ±0.65 × 10^6^ cells), thus balancing the H_2_O_2_ induced-damage (*p* < 0.0001, Figure 9a). Then, we measured telomere length in the ovarian germ cells obtained from the three mice groups in order to investigate a well-established parameter involved in the aging [82,83,84]. Figure 9b reports the analysis of telomeres using a peptide nucleic acid (PNA) fluorescent probe that hybrids the telomere’s repeated sequence. The mean intensity fluorescence is directly related to the repeat number of PNA sequences, which, in turn, is related to telomere length. Ovarian germ cells isolated from the H_2_O_2_ group showed twice lower telomeres’ length (5429.6 ± 534.7 a.u., *p* < 0.0001, SD = ±1690.9 a.u.) than those isolated from the control mice (9898.2 ± 103.6 a.u., SD = ±327.6 a.u.). Ovarian germ cells isolated from the mice group treated with Exocomplex^®^ showed significantly longer telomeres (11,010.4 ± 513.1 a.u., *p* < 0.0001, SD = ±1622.6 a.u.) than mice subjected to oxidative stress only and with measures comparable to untreated mice (Figure 9b). 

The 8-hydroxy-2′-deoxyguanosine (8-OHdG) is the product of the interaction between oxygen-free radicals and the nucleobases of the DNA strand, such as guanine; thus, it is commonly considered a DNA damage marker [85,86] and is associated to carcinogenesis and degenerative diseases [87,88,89]. Thus, we wanted to compare the telomere length of the ovaries to the 8-OHdG serum levels. Figure 9b shows the 8-OHdG serum concentration compared to the telomere length. The results showed that mice treated with Exocomplex^®^ had significantly decreased serum levels of 8-OHdG (1.09 ± 0.13 ng/mL, *p* < 0.0001, SD = ±0.42 ng/mL, Figure 9b) compared to mice exclusively treated with H_2_O_2_ (3.79 ± 0.03 ng/mL, SD = ±0.11 ng/mL), reaching values comparable to the control H_2_O group (1.22 ± 0.22 ng/mL, SD = ±0.71 ng/mL). Figure 9b clearly shows that the Exocomplex^®^-treated group had an inverse behavior of the two parameters; that is, the longer telomers corresponded to reduced DNA damage, suggesting a proper DNA repair. It is known that a tendency to undergo DNA damage affects the fertility [90], and the ability of Exocomplex^®^ to promote DNA repair makes it a potential supplement for female reproductive health preventing fertility complications linked to stress-related events.

#### 3.2.7. Exocomplex^®^ Restores Physiological Levels of Serotonin and Melatonin in Murine Body Fluids

**Figure 10:**
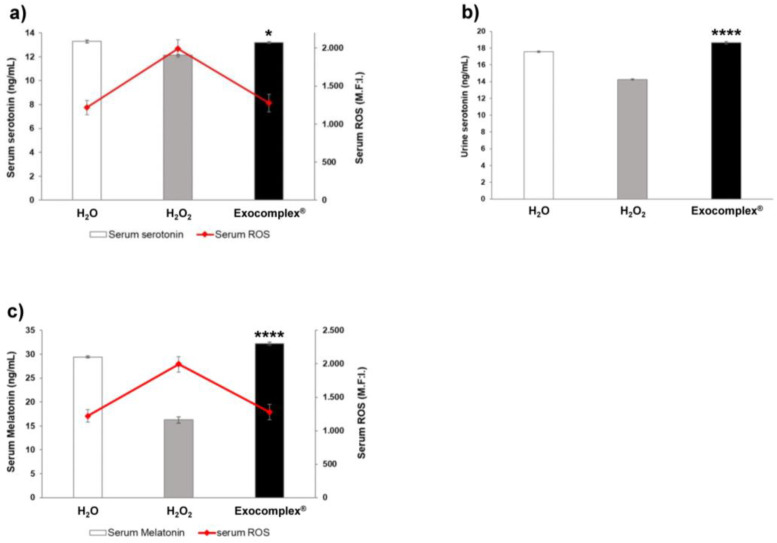
Effect of Exocomplex^®^ treatment on levels of serotonin and melatonin in murine body fluids. (**a**) Comparison between the ROS (M.F.I., a.u.) and serotonin levels (ng/mL) measured in serum of blood samples obtained right before the sacrifice of the mice. Green fluorescence values related to ROS (M.F.I., a.u.) were read at 488 nm in a fluorescence microplate reader. The optical density values related to serotonin were read at 405 nm in a microplate reader, and the serotonin concentration (ng/mL) was calculated from the standard curve. (**b**) Serotonin concentration (ng/mL) in urine samples collected just before the sacrifice of mice. The optical density values related to serotonin were read at 405 nm in a microplate reader, and the serotonin concentration (ng/mL) was calculated from the standard curve. (**c**) Comparison between the ROS (M.F.I., a.u.) and melatonin levels (ng/mL) measured in serum of blood samples obtained right before the sacrifice of the mice. Green fluorescence values related to ROS (M.F.I., a.u.) were read at 488 nm in a fluorescence microplate reader. The optical density values related to serotonin were read at 450 nm in a microplate reader, and the melatonin concentration (ng/mL) was calculated from the standard curve. Data are expressed as means ± SE. * *p* < 0.1, **** *p* < 0.0001.

It is known that a redox imbalance may affect both sleep and circadian rhythm. Among the most known molecules involved in the regulation of the above functions are melatonin and its precursor serotonin [91,92,93,94,95,96,97], which are also known as potent free-radical scavengers and antioxidants [98,99,100,101,102]. Thus, we moved to measure the levels of both melatonin and serotonin in mice body fluids (Figure 10). First, we measured serotonin concentration in the serum and urine of H_2_O_2_-treated mice. The results showed that H_2_O_2_ treatment significantly reduced serotonin both in the serum (12.14 ± 0.07 ng/mL, *p* < 0.0001, SD = ±0.21 ng/mL, Figure 10a) and the urine (14.24 ± 0.06 ng/mL, *p* < 0.0001, SD = ±0.20 ng/mL, Figure 10b), compared to mice that received H_2_O only (13.28 ± 0.12 ng/mL, SD = ±0.40 ng/mL, in serum; 17.56 ± 0.08 ng/mL, SD = ±0.27 ng/mL, in urine). Exocomplex^®^ treatment entirely restored serotonin levels (13.19 ± 0.08 ng/mL, SD = ±0.25 ng/mL, in serum, *p* < 0.05, Figure 10a; 18.63 ± 0.16 ng/mL, SD = ±0.49 ng/mL, in urine, *p* < 0.0001, Figure 10b), with values comparable to those of control mice to basal level. We, thus, measured melatonin serum concentration in the various mice groups. In the H_2_O_2_ group, the serum melatonin was lower (16.22 ± 0.66 ng/mL, *p* < 0.0001, SD = ±2.10 ng/mL, Figure 10c) than in the control group (29.44 ± 0.19 ng/mL, SD = ±0.59 ng/mL), while the Exocomplex^®^ treated group showed a significant increase in the concentration of serum melatonin (32.25 ± 0.33 ng/mL, *p* < 0.0001, SD = ±1.06 ng/mL, Figure 10c) compared to H_2_O_2_ group and leading to values comparable to the group receiving H_2_O only. Figure 10a,c shows a comparison between, respectively, serum serotonin (Figure 10a), melatonin (Figure 10c), and ROS serum levels. Both serotonin and melatonin behaved inversely to ROS in the sera of the mice. In fact, the low serotonin and melatonin serum levels corresponded to the high circulating ROS in the H_2_O_2_-treated group; conversely, the high serotonin and melatonin serum levels corresponded to the low ROS levels in the Exocomplex^®^ treated group. 

These last results definitively showed that all the systemic and organ-related effects induced in vivo by the Exocomplex^®^ were due to a clear anti-oxidant effect that accounted for a complete restoration of the physiological functions in heavily oxidized mice by H_2_O_2_ treatment.

## 4. Discussion

This study was aimed at demonstrating that plant-derived exosomes may be combined to obtain the best anti-oxidant effect not only in preventing diseases but in helping to control disease states either depending on or generating a redox imbalance. To this purpose, we have set up a new compound deriving from the mix of exosomes deriving from different fruits and vegetables, all obtained with organic farming. These include asparagus (*Asparagus officinalis*), cherry (*Prunus avium* L.), grape (*Vitis vinifera* L.), kiwi (*Actinidia chinensis*), orange (*Citrus sinensis* (L.) Osbeck), blood orange (*Citrus sinensis* (L.) Osbeck “Blood Orange”), lemon (*Citrus limon* (L.) Osbeck), mango (*Mangifera indica* L.), papaya (*Carica papaya* L.), grapefruit (*Citrus paradisi Macfad*.), bergamot (Citrus × bergamia Risso and Poit.), tomato (*Solanum lycopersicum* L.). Why have we decided to use exclusively the products of organic farming? We previously identified and characterized edible plant-derived exosomes obtained from fruits of organic agriculture [19]. In that study, we compared exosomes purified from fruits deriving from either organic or conventional farming in which pesticides and microbicides are commonly used [19]. As it has been described for human exosomes [103,104,105], plant-derived exosomes belong to a sort of “scavenging apparatus” that helps all living beings to eliminate toxic and, in general, unwanted material. Thus, intensive agriculture-derived exosomes may concentrate a series of toxic molecules [106,107], making a potentially healthy product poisonous. Organic farming, while not allowing to exclude some environmental contaminants and natural toxins present in the soil, is free from the pesticides and microbicides used for intensive farming. In fact, we excluded the presence of pesticides and microbicides in our PDE mixes (not shown). Moreover, at an equal volume of fruit juice, nanovesicles from organic agriculture juice contain significantly more exosomes and a higher antioxidant content compared to the juice from intensive agriculture [19]. To note, in preliminary experiments, we have compared the total antioxidant capacity (TAC) of PDE to the one exerted by commercially available antioxidants (e.g., ascorbic acid, glutathione), showing significantly more TAC in PDE (not shown). This convinced us not to use commercially available antioxidants in our in vivo experiments due to their low level of antioxidant activity. In this study, we have mixed fruit and vegetable-derived exosomes with the aim of obtaining a final product with the highest antioxidant content. The product was called Exocomplex^®^ and was based on a pool of antioxidants derived from different fruits and vegetables. In fact, here we have shown that in the Exocomplex^®^, the following bioactives are included: catalase; GSH; SOD; ascorbic acid; melatonin; phenolic compounds; and ATP, together with a high total antioxidant capacity (see Table 1). The exosomes contained in the mix were homogeneous in terms of number, size, and distribution, as assessed by NTA. Ultrastructure analysis showed the expected round shape, the full integrity of the PDE, and the unbroken bilayer membrane. We knew that the above bioactives were fully able to scavenge reactive oxygen species (ROS) but also did H_2_O_2_, with a dramatic effect on many cellular processes, including mitochondrial electron transport, β-oxidation of the fatty acids, and photorespiratory oxidation [78,79,108,109,110,111]. To test the in vivo effects of our PDE mixes, we used a model of mice treated for two weeks with H_2_O_2_ in order to induce oxidative damage at the organ level. After that, we treated the mice with Exocomplex^®^ for 3 weeks and analyzed oxidative markers in murine sera, comparing mice treated with H_2_O_2_ alone to those treated with H_2_O_2_ and Exocomplex^®^, to mice left untreated. 

The first result was a relevant and significant decrease in plasmatic ROS levels in mice treated with Exocomplex^®^, with levels comparable to control mice compared to mice treated with H_2_O_2_ only. Malondialdehyde (MDA) is one of the final products of polyunsaturated fatty acids peroxidation, and higher production of MDA is correlated to increased ROS concentration. Thus, in the same mice, we analyzed the serum MDA, finding that, consistent with the decreased ROS levels, mice treated with Exocomplex^®^ showed a significant decrease in MDA serum concentration. To further support these systemic data, we measured the serum levels of 8-hydroxy-2′-deoxyguanosine (8-OHdG), which is commonly considered a DNA damage marker. The results showed that mice treated with Exocomplex^®^ had a marked and significant decrease in 8-OHdG serum concentration compared to mice subjected to oxidative stress. With this set of results, we showed that Exocomplex^®^ oral treatment produced a systemic anti-oxidant response leading to a reduction in ROS and lipid peroxidation levels, together with significant inhibition of DNA damage. This is actually the first in vivo evidence that a natural product, such as the mix of exosomes deriving from the products of organic farming that we called Exocomplex^®^, is able to lead to a full recovery after the induction of a heavy state of intoxication, as it has been reproduced by a long-standing administration of H_2_O_2_ in the daily water. This positive response was induced by a clear systemic anti-oxidant response that reduced both ROS, MDA, and DNA damage. However, we did not know whether the restored redox balance positively affected some cellular functions throughout the body of the Exocomplex^®^-treated mice. Thus, we explored the effect of oral treatment with Exocomplex^®^ in restoring cellular proliferation in the bone marrow and the spleen of H_2_O_2_-treated mice. The results showed a marked reduction in cells isolated from either bone marrow or spleen in H_2_O_2_-treated mice compared to untreated mice, while mice subjected to oxidative stress and then treated with Exocomplex^®^ showed a significant increase in the number of both bone marrow cells and splenocytes compared to mice exclusively treated with H_2_O_2_. These results further supported a clear effect of Exocomplex^®^ treatment in restoring the reduction in cells induced by the H_2_O_2_-oxidative stress. In fact, the values found in the Exocomplex^®^-treated mice were fully comparable to the values of the mice that received only water from the start of the experiment. The results obtained with the cell count were consistent with the proliferation rates of the same cells in the various organs. Moreover, the Exocomplex^®^ treatment, together with reducing the serum ROS levels, entirely recovered the mitochondrial membrane potential and mitochondrial superoxide induced by H_2_O_2_ treatment, reaching levels comparable to the control mice. We know that mitochondrial ROS promote cellular senescence and contribute to aging, by promoting mitochondrial oxidative stress [7,77], in turn suggesting that treatment with Exocomplex^®^ could be used in counteracting aging. 

We found that the immunoglobulins serum levels in mice undergoing H_2_O_2_-induced oxidative stress were dramatically reduced. Thus, we evaluated the effect of the oral treatment with Exocomplex^®^ on the plasmatic immunoglobulin levels. We found that mice treated with Exocomplex^®^ following the oxidative stress had a significant increase in the serum immunoglobulins levels, supporting a clear effect of the treatment on the immune response. 

We, thus, moved to investigate the effects of Exocomplex^®^ on murine ovarian germ cells after the H_2_O_2_-induced oxidative stress. H_2_O_2_ treatment induced a dramatic reduction in ovarian germ cells, while Exocomplex^®^ administration entirely recovered the ovarian cell count to a level comparable to that of the control mice. Telomere length is a molecular hallmark related to both aging and oxidative stress. We, thus, evaluated telomere length in single-cell suspensions of ovarian germ cells in the various mice groups. The results showed that ovarian germ cells isolated from H_2_O_2_-treated mice had half the telomeres’ length compared to untreated mice, while telomeres isolated from Exocomplex^®^-treated mice showed significantly longer telomeres, with measures comparable to those of control mice.

The last point we investigated was the ability of our Exocomplex^®^ to modulate the level of molecules related to the circadian rhythms of the body, such as melatonin and its precursor serotonin (proven potent free-radical scavenging and antioxidants). We, thus, measured the levels of melatonin and serotonin in the serum and urine of the various groups of mice. The results showed a significant reduction in serotonin concentration in the serum and urine of H_2_O_2_-treated mice, while Exocomplex^®^ oral treatment restored serotonin concentration in both the serum and urine of treated mice. Comparably to serotonin, melatonin concentration in the serum of H_2_O_2_-treated mice was significantly lower than in mice receiving water only. Treatment with Exocomplex^®^ induced a significant increase in serum melatonin levels, suggesting that Exocomplex^®^ treatment brought serotonin and melatonin levels to levels comparable to the untreated mice. Of interest, both serotonin and melatonin levels behaved inversely to the ROS levels in the sera of the various mice.

## 5. Conclusions

In summary, our results strongly support a clear effect of Exocomplex^®^ in recovering from the H_2_O_2_-mediated damage and redox imbalance. We want to emphasize that the results of our study pioneer the use of a mix of plant-derived exosomes with the purpose of setting up a highly bio-available natural compound with a “super-antioxidant” effect. With this study, we support a strategy through which an Exocomplex^®^ may be built with the idea of targeting different functions, including either a general anti-oxidant reaction or an effect on the circadian rhythms. We want to emphasize again that this may be induced through natural products, with no side effects and no waste (all the waste in the production is reutilized as compost for organic farming). The Exocomplex^®^-based products have the potential to represent a new class of suppliers characterized by a high level of bioavailability, not necessarily allowing the use of high amounts of bioactives to obtain a visible effect. This can be obtained via an oral administration without needing to use an IV or other more invasive routes of administration.

For the first time, we show that a mix of plant-derived exosome-like nanovesicles containing powerful anti-oxidant bioactives may be helpful in both preventing pathological conditions sustained by a redox imbalance and in controlling conditions with an established redox imbalance, which is known to be closely associated with the etiology of many diseases.

Lastly, an effective role of exosomes in modifying the microenvironment has been proposed, which on the one hand, may lead to negative consequences [112], but in the case of plant-derived exosomes, it may lead to a positive reprogramming at the level of virtually all tissues.

## Figures and Tables

**Table 1:** Quantification of bioactive compounds in Exocomplex^®^. Values were normalized per dose of plant-derived exosomes (PDE), 6 × 10^9^/mouse (2 × 10^8^/g mouse weight), administered to the mice.

	Mean	Std Err
SOD (U/mL)	557	13
GSH (µM)	552	12
CATALASE (mU/mL)	1713	30
ASCORBIC ACID (µg)	32	0.01
MELATONIN (ng)	1.81	0.02
TOTAL ANTIOXIDANT CAPACITY (mM)	620.5	27.8
PHENOLIC COMPOUNDS (mM)	769.3	49.1
ATP (µM)	81.6	8.6

## Data Availability

The data presented in this study are available on request from the corresponding author.
